# The First Example of a *Model* Amphiphilic Polymer Conetwork Containing a Hydrophobic Oligopeptide: The Case of End-Linked Tetra[Poly(ethylene glycol)-*b*-oligo(*L*-alanine)]

**DOI:** 10.3390/gels11050331

**Published:** 2025-04-29

**Authors:** Demetris E. Apostolides, George Michael, Costas S. Patrickios, Takamasa Sakai, Iro Kyroglou, Maria Kasimatis, Hermis Iatrou, Sylvain Prévost, Michael Gradzielski

**Affiliations:** 1 Department of Chemistry, University of Cyprus, P.O. Box 20537, Nicosia 1678, Cyprus; apostolides.demetris@ucy.ac.cy (D.E.A.); giorgosmichael296@gmail.com (G.M.); 2 Department of Bioengineering, Graduate School of Engineering, The University of Tokyo, 7-3-1 Hongo, Bunkyo-ku, Tokyo 113-8656, Japan; sakai@tetrapod.t.u-tokyo.ac.jp; 3 Department of Chemistry, National and Kapodistrian University of Athens, Zografou, 15771 Athens, Greece; irokrg@gmail.com (I.K.); mkasimatis@hotmail.gr (M.K.); iatrou@chem.uoa.gr (H.I.); 4 Institut Max von Laue—Paul Langevin (ILL), 71 Avenue des Martyrs—CS 20156, 38042 Grenoble, France; prevost@ill.eu; 5 Stranski-Laboratorium für Physikalische und Theoretische Chemie, Institut für Chemie, Technische Universität Berlin, Straße des 17. Juni 124, 10623 Berlin, Germany; michael.gradzielski@tu-berlin.de

**Keywords:** amphiphilic polymer conetworks, peptides, tetraPEG, SANS, aqueous swelling

## Abstract

Herein we describe the development of the first *model* amphiphilic polymer conetwork (APCN) comprising a short hydrophobic hexa(*L*-alanine) segment being the outer block of an amphiphilic four-armed star block copolymer with inner poly(ethylene glycol) (PEG) blocks bearing benzaldehyde terminal groups and end-linked with another four-armed star PEG homopolymer (tetraPEG star) bearing aryl-substituted acylhydrazide terminal groups. The present successful synthesis that yielded the peptide-containing model APCN was preceded by several unsuccessful efforts that followed different synthetic strategies. In addition to the synthetic work, we also present the structural characterization of the peptide-bearing APCN in D_2_O using small-angle neutron scattering (SANS).

## 1. Introduction

Polymer networks represent an important type of material in human civilization, with their first encounter in recorded history dating back more than 3500 years ago to indigenous populations in North and Central America who used plant extracts in order to crosslink tree-extracted *cis*-1,4-polyisoprene and obtain the first-ever rubber, which was used to make waterproof boots and bouncy toy balls [1]. Nowadays, the four classes of polymer networks—elastomers (rubbers), thermosetting polymers (thermosets), thermoplastic elastomers, and polymer gels—constitute a rapidly growing global market that totaled about 250 billion euros in 2018, representing about one-quarter of the overall global synthetic polymer market for 2018 [2]. A particular type of synthetic polymer gel is amphiphilic polymer conetworks (APCNs), which first appeared in the academic literature in 1988 [3,4,5].

APCNs [6,7,8,9] are crosslinked polymer networks comprising both hydrophilic and hydrophobic polymer segments. This constitution drives APCNs to nanophase separation both in water and in bulk. Thus, these intriguing materials represent the network analogue to surfactants. APCNs are being evaluated for several emerging applications. These include their use as matrices for drug delivery [10], scaffolds for tissue engineering [11], conducting electrode-separating membranes in lithium-ion batteries [12], and biphasic supports for reactions in organic solvents taking place in the apolar nanophase catalyzed by an enzyme entrapped in the polar nanophase [13,14,15]. However, APCNs have one large market application with an annual global value of 10 billion euros: silicone hydrogel soft contact lenses [16,17]. In this type of contact lens, both the hydrophobic and (hydrated) hydrophilic components are soft, offering comfort to the eye. Moreover, nanophase separation of silicone hydrogel soft contact lenses into a bicontinuous gyroid-like morphology secures on-eye lens movement, providing further eye comfort.

Most APCN syntheses are performed via the hydrophobic macro-crosslinker approach [6,7,8], in which a hydrophilic vinyl monomer is randomly copolymerized with a hydrophobic divinyl polymeric or oligomeric crosslinker to yield an APCN comprising well-defined hydrophobic segments randomly interconnecting two different hydrophilic polymer chains. While this is a very convenient synthetic strategy that can be performed either using conventional free radical polymerization or, more recently, controlled radical polymerization [18,19,20], it cannot provide model conetwork structures because, although the length of the hydrophobic polymer segments between crosslinking points is precisely fixed, this is not the case with the length of the hydrophilic segments. Relatively recent efforts by us and other researchers have aimed at making as perfect APCNs as possible via the use of well-defined polymeric building blocks interconnected at their ends [21,22,23,24,25]. The resulting (near-)perfect conetwork structures are important both because they will lend themselves to the derivation of accurate structure–property relationships (upon characterization of the APCN properties) and because they are expected to result in conetworks with improved properties. One such property is the mechanical strength and extensibility, which will be improved because, with all chains having the same length, there will be no early breaking of shorter chains upon elongation.

Whether model or not, the driving force for APCN nanophase separation in the aqueous environment is the nature of hydrophobic segments. Both mildly hydrophobic and more strongly hydrophobic segments have been employed for APCN synthesis. Regarding the former, poly(propylene glycol) (PPG) is a representative example, in which its etheric main-chain-polymer oxygen renders it rather polar (and, consequently, mildly hydrophobic) and temperature-sensitive (with hydrophobicity increasing with an increase in temperature in water). PPG is most conveniently introduced if the APCNs comprise amphiphilic Pluronics or Tetronics whose hydrophobic component is already PPG [23,25,26,27,28,29]. Regarding more hydrophobic segments used in APCNs, these have included polystyrene [12,30], polydimethylsiloxane (PDMS) [31,32,33], poly(*ε*-caprolactone) (PCL) [22,24,34], poly(2-ethylhexyl acrylate) [35], poly(2-*n*-butyloxazoline) [36], polyisobutylene (PIB) [37,38], poly(2-(1-ethylpentyl)-2-oxazoline) [39], and hydrophobic peptides such as poly(*β*-benzyl-*L*-aspartate) [40]. The incorporation of hydrophobic peptides in APCNs is very rare because of the difficulty with peptide preparation and peptide attachment onto the network. However, it may be very beneficial for the APCN properties because, in the above-mentioned recent example [40], the mechanical properties of the produced APCNs were highly enhanced as a result of, primarily, intra-peptide structural organization within the APCN. Consequently, the APCNs in reference [40], as well as the presently developed APCN, may find applications as biomedical implants, wound coverings, and breathable membranes in textiles where toughness is required.

The purpose of the present investigation is to build on the above example, incorporating a hydrophobic peptide in the APCN. The particular objective is to include the peptide not as a hydrophobic macro-crosslinker, which would impart randomness to the APCN structure, as was the case in the above-mentioned work [40], but rather to ensure that the APCN structure is as perfect as possible. To this end, an appropriately end-functionalized four-armed hydrophilic star homopolymer was used as a macroinitiator to grow short segments of a hydrophobic peptide off the star’s four termini. This strategy of amphiphilic star block copolymer preparation proved much more efficient than our previous efforts to attach a separately prepared end-functionalized hydrophobic linear peptide onto a complementarily end-functionalized hydrophilic star polymer. Subsequently, the obtained amphiphilic star block copolymer was further end-functionalized so as to render its four termini reactive to the functional (reactive) groups of the chosen crosslinker. This latter end-functionalization strategy was taken from the peptide literature and was more direct and more efficient than other more conventional synthetic organic chemistry strategies that also required protection and deprotection steps. The present communication is mainly synthetic, reporting our long-sought preparation of a peptide-containing model APCN. Yet, we were able to also examine the aqueous self-assembling properties of the presently prepared peptide APCN using small-angle neutron scattering (SANS).

## 2. Results and Discussion

**Figure 1:**
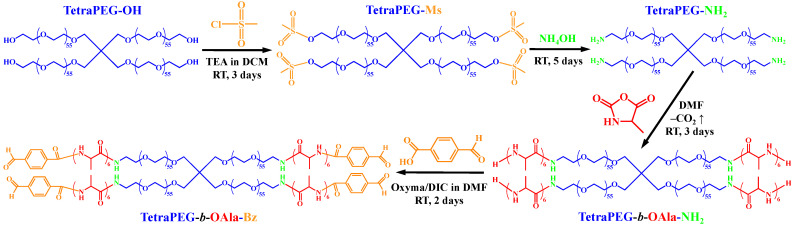
Reaction scheme leading to the four-step transformation of the tetraPEG-OH star into the tetraPEG-*b*-OAla-Bz star APCN building block.

Synthetic Strategy for the Main Peptide-Containing Polymeric Building Block. Figure 1 presents the reaction scheme illustrating the four-step transformation of the starting tetrahydroxy-terminated four-arm star poly(ethylene glycol) (tetraPEG-OH star) into the star block copolymer bearing oligo(*L*-alanine) outer segments and reactive terminal benzaldehyde moieties. The first step involves the activation of the hydroxyl end-groups via mesylation through the reaction of the tetraPEG-OH star with mesyl chloride in dichloromethane (DCM) in the presence of triethylamine (TEA) to obtain the tetraPEG-Ms star at a 90% yield. This is followed by aminolysis of the mesyl terminal groups in the tetraPEG-Ms star to convert them to primary amine groups and produce the tetraPEG-NH_2_ star at a 72% yield. Next, the tetraPEG-NH_2_ star is employed as a macroinitiator to oligomerize via ring-opening polymerization in *N*,*N*-dimethylformamide (DMF), with the *L*-alanine *N*-carboxy anhydride (Ala-NCA) off the macroinitiator’s primary amine end-groups in order to obtain the tetraPEG-*b*-OAla-NH_2_ star. Finally, the primary amine terminal groups of the above-mentioned star block copolymer are conjugated through an oxyma/DIC-catalyzed amidation reaction [41,42] with 4-formylbenzoic acid in DMF to install a benzaldehyde moiety at the terminus of each arm of the star block copolymer and obtain the tetraPEG-*b*-OAla-Bz star (51% yield after dialysis). These terminal benzaldehyde groups of the amphiphilic tetraPEG-*b*-OAla-Bz star block copolymer are reactive to the terminal aryl-substituted acylhydrazide groups of the hydrophilic tetraPEG star homopolymer, the tetraPEG-Hz star, so that a combination of these two star polymers would yield the desired peptide-containing model APCN.

**Figure 2:**
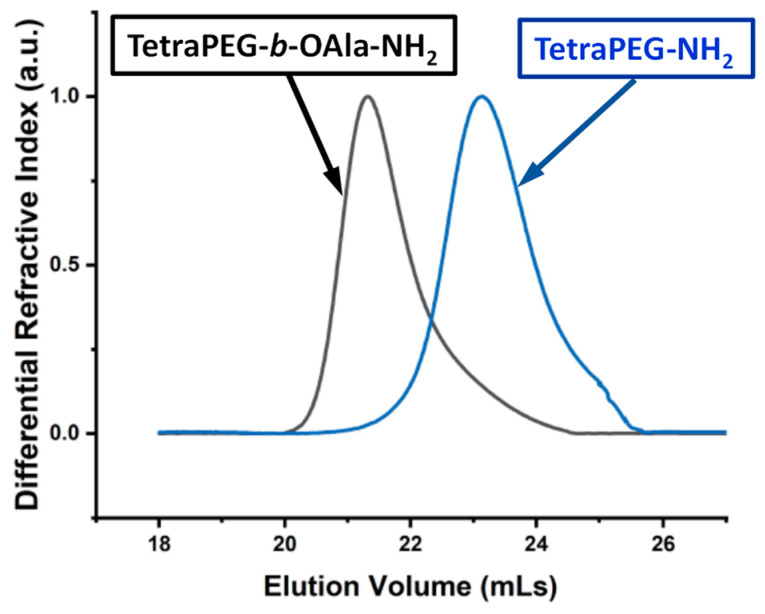
Size exclusion chromatograms of the tetraPEG-NH_2_ and tetraPEG-*b*-OAla-NH_2_ star polymers.

Size Exclusion Chromatography. Size exclusion chromatography (SEC) was employed to confirm the growth of the *L*-alanine monomer repeating units off the primary-amine termini of the tetraPEG-NH_2_ star hydrophilic homopolymer with the conversion of this polymer into an amphiphilic star block copolymer with short oligo(*L*-alanine) outer blocks. Figure 2 illustrates the SEC traces of these two star polymers. The figure indicates the expected growth of the tetraPEG-NH_2_ star homopolymer precursor to a larger polymer, tetraPEG-*b*-OAla-NH_2_, clearly eluting earlier than its precursor homopolymer; a smaller elution volume is consistent with a larger polymer molar mass. Furthermore, the size exclusion chromatogram of the tetraPEG-*b*-OAla-NH_2_ star block copolymer product possesses a slight tailing toward higher elution volumes (smaller molar masses), apparently originating from the parent tetraPEG-NH_2_ star homopolymer. The (relative) number-average molar masses, *M*_n_, and molar mass distributions, *Ð*, for the two star polymers were calculated from the SEC traces in Figure 2 and are listed in Table 1. The table also lists the theoretical molar masses, *MM*_theory_, for the two star polymers in order to compare them with the corresponding experimental (*M*_n_) values from SEC.

Table 1 shows that the *M*_n_ value for the tetraPEG-*b*-OAla-NH_2_ star block copolymer product is higher than that of its tetraPEG-NH_2_ star homopolymer precursor, as expected. Moreover, both *M*_n_ values are close to the theoretically estimated *MM*_theory_ values, although both former values are higher than the latter by about 1000 g mol^–1^. However, the increase in the *M*_n_ value from that of the precursor star homopolymer to that of the star block copolymer product is 1800 g mol^–1^, very close to 1704 g mol^–1^, the value corresponding to the 24 *L*-alanine monomer repeating units added to the star polymer (six *L*-alanine monomer repeating units per arm × four arms) times the value of the molar mass of the *L*-alanine monomer repeating unit of 71 g mol^–1^. Finally, the *Ð* values of the two star polymers are relatively low, at about 1.1, despite the tailing in their SEC traces.

**Figure 3:**
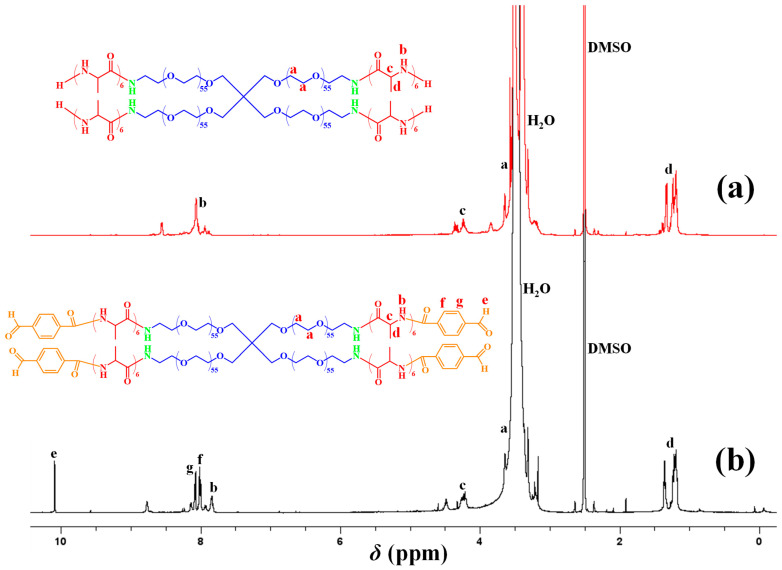
^1^H NMR spectra in *d*_6_-DMSO of the (**a**) tetraPEG-*b*-OAla-NH_2_ star block copolymer and (**b**) its end-functionalized derivative tetraPEG-*b*-OAla-Bz. The peaks labeled DMSO and H_2_O at 2.5 and 3.5 ppm, respectively, are due to the hydrogenated impurities in the deuterated solvent.

^1^H NMR Spectroscopy. Figure 3 shows the composition characterization of the tetraPEG-*b*-OAla-Bz star peptide-bearing building block (part (b) of Figure 3) and its tetraPEG-*b*-OAla-NH_2_ precursor (part (a)) using ^1^H NMR spectroscopy. The ^1^H NMR spectra indicate the incorporation of *L*-alanine monomer repeating units (peaks b, c, and d) and benzaldehyde end-groups (peaks e, f, and g). Considering the relative peak areas in the ^1^H NMR spectra, it was determined that six *L*-alanine monomer repeating units were introduced per arm, whereas each arm was essentially fully end-functionalized with the benzaldehyde moiety.

**Figure 4:**
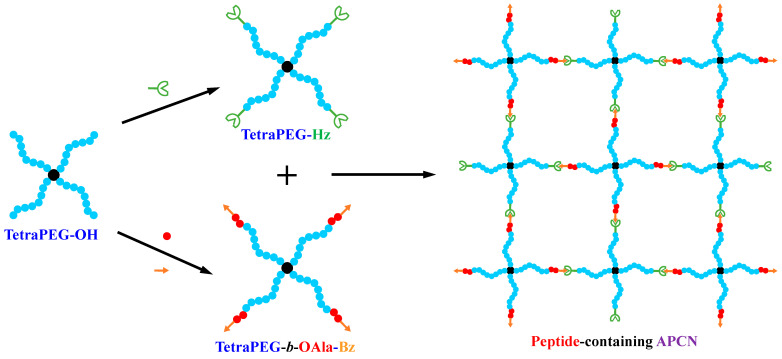
Modification of the tetraPEG-OH star starting polymer to obtain the two main polymeric building blocks, the tetraPEG-*b*-OAla-Bz end-functionalized amphiphilic star block copolymer and the tetraPEG-Hz end-functionalized hydrophilic star homopolymer, and their end-linking to obtain the desired *model* APCN.

APCN Synthesis Scheme. Figure 4 diagrammatically illustrates the formation of the peptide-bearing *model* APCN from the end-linking of its two star polymer building blocks and the prior preparation of these building blocks from the tetraPEG-OH star homopolymer starting material via its end-functionalizations and also a ring-opening polymerization step. Drawing a perfect APCN structure in Figure 4 assumes a perfect structure of the star polymer building blocks and their quantitative end-linking to a model network. Regarding the former assumption, this is largely supported by the tetraPEG-OH manufacturer’s (NOF Corporation) detailed characterization of this material, which indicated that it bears exactly four arms, with all star arms having the same length, and the overall star polymer having a molar mass dispersity of about 1.1. Our SEC traces and ^1^H NMR spectra strongly suggest that this homogeneity was preserved upon the growth of the oligo(*L*-alanine) short segment. Furthermore, ^1^H NMR spectra also strongly suggest that the end-functionalizations of the tetraPEG-OH star homopolymer and the tetraPEG-*b*-OAla-NH_2_ with benzaacylhydrazide and benzaldehyde groups, respectively, were quantitative. Regarding the latter assumption, it is supported by indirect evidence. This indirect evidence is the quantitative end-functionalization of the star polymer building blocks from ^1^H NMR spectroscopy (see above) and the fact that a gel was formed, exhibiting an aqueous swelling degree with the expected value. Even more indirect evidence of the quantitative end-linking between the star components comes from some of our previous work where the same end-linking reaction was conducted in water (components were more hydrophilic) and where ^1^H NMR spectroscopy performed in D_2_O during the gel formation allowed the determination of an 85% or higher conversion of the crosslinking reaction [23].

Equilibrium Degree of Swelling in Water. The APCN was transferred from DMF, the gel formation solvent, to water. The supernatant water was replaced every day for seven days to secure the complete removal of DMF. This was also confirmed using ^1^H NMR spectroscopy. Subsequently, the water content in the peptide-containing APCN was determined gravimetrically, and from that, the APCN aqueous swelling degree was calculated to be equal to 11. This value is expectedly lower (due to the incorporation of the hydrophobic peptide in this amphiphilic gel sample) than the corresponding value for the tetraPEG homopolymer gel prepared using tetraPEG stars of the same molar mass and interlinked via the same chemistry, which was measured to be equal to 16 [43].

**Figure 5:**
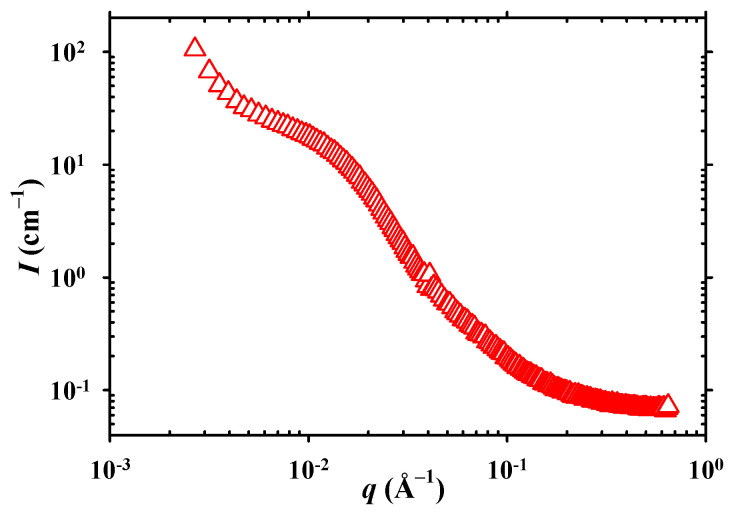
SANS profile of the peptide-containing APCN in D_2_O.

SANS. Figure 5 plots the SANS profile of the final peptide-bearing APCN in D_2_O. The SANS profile presents only a shoulder rather than a clear correlation peak, as the hydrophobic oligo(*L*-alanine) segment is not sufficiently long enough to strongly drive its self-association and yield a well-defined hydrophobic nanophase in D_2_O. Thus, the association among oligo(*L*-alanine) blocks must be limited, if there is any at all. The location of the shoulder at 0.012 Å^–1^ corresponds to a distance, *d*, of 52.3 nm, calculated as *d* = 2π/*q_shoulder_* = 6.28/0.012 Å = 523.3 Å. This distance is even slightly longer than the contour length between two consecutive oligo(*L*-alanine) segments, estimated as 47.4 nm {= [2 × (56 + 6 + 2) × 0.37 nm = 47.36 nm], where 56 and 6 are, respectively, the number of ethylene glycol and *L*-alanine monomer repeating units in one arm, whereas the size of the arm’s aromatic end-group is assumed to correspond to two monomer repeating units; the contribution of each monomer repeating unit to the contour length is taken to be 0.37 nm}, suggesting that the shoulder should not correspond to any proper hydrophobic aggregation.

## 3. Conclusions

The protocol for the successful preparation of model (near-perfect) amphiphilic polymer conetworks (APCNs) based on two complementarily end-functionalized tetraPEG stars, one of which also contains a hydrophobic peptide component, is presented in this report. In developing this protocol, difficulties were encountered when we attempted to attach a separately prepared hydrophobic peptide onto the tetraPEG star and when we tried to end-functionalize the peptide. The former problem was solved by directly polymerizing the cyclized amino acid monomer off the primary-amine-functionalized termini of the tetraPEG star, while the latter challenge was addressed by appropriately end-functionalizing the peptide terminus using the very efficient “Oxyma” linking chemistry. The relatively small amounts of the polymeric materials available and the several unsuccessful preliminary synthetic efforts did not allow for APCN property characterization in this preliminary work, other than some aqueous swelling and SANS measurements. Importantly, however, the synthetic proof of concept was achieved. Future work will include APCN mechanical property characterization, preferably on APCN samples bearing longer hydrophobic peptides and even peptides based on amino acids of various hydrophobicities.

## 4. Experimental Section

### 4.1. Materials

*N*,*N*-dimethylformamide (DMF, 99.8%), triethylamine (TEA, ≥99.5%), methanesulfonyl chloride (≥99.7%), dichloromethane (≥99.8%, DCM, CH_2_Cl_2_), ammonium hydroxide (NH_4_OH) aqueous solution (80% *w*/*w*), anhydrous magnesium sulfate (≥99.5%, MgSO_4_), sodium hydrogen carbonate (NaHCO_3_, ≥99.7%), sodium hydroxide (NaOH), calcium hydride (CaH_2_, 90–95%), ethyl (2*Z*)-2-cyano-2-(hydroxyimino)acetate (“Oxyma”, ≥99%), *N*,*N*′-diisopropylcarbodiimide (99%, DIC), dialysis tubing composed of benzoylated cellulose with a molar mass cut-off of 3000 g mol^−1^ (average flat width 32 mm), 1,4-dioxane (≥99%), diethyl ether (≥99.5%, C_2_H_5_OC_2_H_5_), 4-formylbenzoic acid (≥98%), deuterated chloroform (CDCl_3_, 99.8%), and deuterated dimethyl sulfoxide (*d*_6_-DMSO, 99.8%) were purchased from Sigma-Aldrich-Merck, Darmstadt, Germany. Tetrahydroxy-terminated four-arm star poly(ethylene glycol) of *M*_n_ = 10,000 g mol^−1^ (tetraPEG-OH) was purchased from NOF Corporation, Tokyo, Japan. Tetraacylhydrazide-terminated four-arm star poly(ethylene glycol) of *M*_n_ = 10,536 g mol^−1^ (tetraPEG-Hz) was prepared from tetraPEG-OH according to our previously published procedure [43].

### 4.2. Synthetic Methods

Synthesis of the tetramesyl-terminated four-arm star poly(ethylene glycol) of *M*_n_ = 10,312 g mol^−1^ (tetraPEG-Ms). TetraPEG-OH (7.0 g, 0.7 mmol) was dried by first being dissolved in anhydrous 1,4-dioxane and then being freeze-dried. Afterward, the dried tetraPEG-OH was transferred into a round-bottomed flask and dissolved in 40 mL of dried DCM that had been freshly distilled from CaH_2_. Then, dried TEA (2.7 mL, 2.0 g, 19.6 mmol), also freshly distilled from CaH_2_, was added into the polymer solution in DCM. After that, the mixture was cooled down to 0 °C in an ice bath, and methanesulfonyl chloride (1.52 mL, 2.24 g, 19.6 mmol) was added dropwise under stirring. The stirred reaction mixture was left for 48 h and was subsequently diluted with DCM and washed with 150 mL of a 10% *w*/*v* aqueous solution of NaHCO_3_ (6 times, 25 mL each time) and with 100 mL of water (4 times, 25 mL each time). Next, the mixture was dried with anhydrous magnesium sulfate, filtered, and concentrated under reduced pressure using a rotary evaporator. Afterward, the resulting solution was precipitated in cold diethyl ether twice. Then, the final precipitate was dried under vacuum at 45 °C, and the resulting tetraPEG-Ms product was isolated as a white powder at a 90% yield (6.5 g, 0.63 mmol).

Synthesis of the tetraamine-terminated four-arm star poly(ethylene glycol) of *M*_n_ = 9996 g mol^−1^ (tetraPEG-NH_2_). First, tetraPEG-Ms (5.60 g, 0.543 mmol) was transferred into a round-bottomed flask and dissolved in 84 mL of an 80% *w*/*w* ammonium hydroxide aqueous solution. Then, the flask was sealed, and the mixture was left under stirring for five days. Afterward, the mixture was concentrated under reduced pressure using the rotary evaporator, and it was then diluted with 150 mL of a 1 M aqueous solution of sodium hydroxide. Subsequently, the aqueous reaction mixture was washed with 200 mL of DCM (8 times, 25 mL each). Then, the organic phases were combined, and the mixture was concentrated under reduced pressure using the rotary evaporator. After that, the resulting solution was precipitated in cold diethyl ether. The precipitate was dried under vacuum at 45 °C, and the resulting tetraPEG-NH_2_ product was isolated as a white powder at a 72% yield (3.9 g, 0.39 mmol).

Synthesis of the *N*-carboxy anhydride (NCA) of *L*-alanine (Ala-NCA). The synthesis of the *N*-carboxy anhydride (NCA) of alanine (Ala-NCA) was accomplished following our previously published approach [44]. According to that approach, Ala-NCA was synthesized from the corresponding *L*-*α*-amino acid and triphosgene in a suspension of acetonitrile at 70 °C under an inert atmosphere. The unreacted species, along with the amino acid salts (insoluble species), were removed by filtration. Ala-NCA was subsequently dissolved and dried by distilling off the solvent several times with ethyl acetate under a high vacuum in order to remove the excess triphosgene, which sublimes under a high vacuum, along with the remaining HCl. Finally, Ala-NCA was dissolved in ethyl acetate and recrystallized from *n*-hexane three times under high vacuum in a custom-made apparatus. The purity of Ala-NCA was confirmed using ^1^H NMR spectroscopy in CDCl_3_ and FT-IR spectroscopy. Both of these spectra are illustrated in the Supporting Materials. The ^1^H NMR spectrum of Ala-NCA displayed only the three expected peaks of the sample (plus the solvent peak at 7.23 ppm due to the remaining hydrogenated CHCl_3_ impurity in CDCl_3_). On the other hand, the FT-IR spectrum of Ala-NCA possessed no signal from a peptide bond at 1640 cm^−1^, a frequent impurity in amino acid anhydrides. The purified NCA was stored under an inert atmosphere at 0 °C. The yield was 89%.

Synthesis of the tetraamine-terminated four-arm poly(ethylene glycol)-*b*-oligo(L-alanine) star block copolymer (tetraPEG-*b*-OAla-NH_2_). Dry tetraPEG-NH_2_ was placed in a custom-made glass reactor equipped with a high-vacuum stopcock. The polymer was further dried for one night under a high vacuum, followed by distillation of dry benzene. After dissolving the polymer, benzene was distilled off to dryness. This way, the polymer was dried to the extent required for the ring-opening polymerization of Ala-NCA. Then, dry DMF was distilled into the reactor to dissolve the tetraPEG-NH_2_ polymeric macroinitiator, followed by the addition of a solution of Ala-NCA also in dry DMF through an ampoule by rupturing the break-seal. The amount of Ala-NCA was calculated so that six L-alanine monomeric repeating units could be added to each arm. The polymerization lasted 3 days, and the solution was evacuated from time to time to remove the produced CO_2_. Then, the resulting tetraPEG-*b*-OAla-NH_2_ star block polymer product was precipitated in n-hexane, dried, and stored at −20 °C.

Synthesis of tetrabenzaldehyde-terminated four-arm star poly(ethylene glycol)-*b*-oligo(L-alanine) block copolymer (tetraPEG-*b*-OAla-Bz). TetraPEG-*b*-OAla-NH_2_ star block copolymer (0.1 g, 9.2 μmol), ethyl (2*Z*)-2-cyano-2-(hydroxyimino)acetate (“Oxyma”, 0.08 g, 0.55 mmol), and 4-formylbenzoic acid (0.08 g, 0.55 mmol) were all transferred into a round-bottomed flask and dissolved in freshly distilled DMF (4 mL). Then, DIC (88 μL, 0.070 g, 0.55 mmol) was added dropwise into the mixture under stirring. After 2 days, the reaction mixture was transferred into a benzoylated cellulose membrane in which the mixture was dialyzed against DMF for 3 days, with the external DMF being changed every day. Then, the mixture was dialyzed against 1,4-dioxane for 2 days, with the external 1,4-dioxane being changed every day. Afterward, the dialyzed solution was freeze-dried, and the tetraPEG-*b*-OAla-Bz was isolated as a white powder at a 51% yield (0.054 g, 4.62 μmol).

Synthesis of the tetraPEG-Hz—tetraPEG-*b*-OAla-Bz amphiphilic polymer conetwork (APCN). The amphiphilic polymer conetwork (APCN) was prepared by mixing DMF solutions of the homopolymer four-arm star tetraPEG-Hz and the tetraPEG-*b*-OAla-Bz amphiphilic star block copolymer at their stoichiometric molar ratio and at room temperature, with a total polymer concentration equal to 10.0% *w*/*v*. To this end, 0.0137 g (1.3 μmol) of the tetraPEG-Hz and 0.0157 g (1.3 μmol) of the tetraPEG-*b*-OAla-Bz were dissolved separately, each in 150 μL of DMF. Then, 2 μL of acetic acid was added into the tetraPEG-*b*-OAla-Bz solution to act as a catalyst. Subsequently, the tetraPEG-Hz and tetraPEG-*b*-OAla-Bz solutions were mixed, and the mixture was left at room temperature for one week for gel formation. Finally, the formed gel was transferred from DMF into water. Total DMF removal from the gel was attained by changing the supernatant water every day for seven days.

### 4.3. Characterization Methods

Degree of Swelling. The degree of swelling of the APCN in water was determined gravimetrically after exchange from DMF and equilibration in water for one week by changing the water daily. The process of solvent exchange involves the direct transfer of the gel from DMF to water, followed by total DMF removal by renewing the water several times rather than removing all DMF by vacuum-drying and then swelling in water.

Size Exclusion Chromatography (SEC). The number-average molar masses, *M*_n_, and molar mass distributions, *Ð* = *M*_w_/*M*_n_, of the homopolymer star macroinitiator, TetraPEG-NH_2_, and the amphiphilic star block copolymer, TetraPEG-*b*-OAla-NH_2_, were determined using size exclusion chromatography (SEC) on a Waters high-performance liquid chromatograph. This was equipped with a Waters 600 pump, four Waters Ultrastyragel SEC columns (HT-2, HT-4, HT-5E, and HT-6E), a Waters 410 differential refractometer, and a two-angle (15° and 90°) Precision PD 2020 light scattering detector operated at 60 °C. The eluent was a 0.1 M LiBr solution in DMF delivered at a rate of 1 mL min^−1^.

^1^H NMR Spectroscopy. The chemical structures of the end-functionalized star polymers were characterized using ^1^H NMR spectroscopy on a 500 MHz Bruker spectrometer in *d*_6_-DMSO.

Small-Angle Neutron Scattering (SANS). The following sample preparation was conducted in order to perform small-angle neutron scattering (SANS) on the APCN. The H_2_O-equilibrated APCN was thoroughly vacuum-dried and transferred to a quartz cuvette, where it was equilibrated in D_2_O. The swelling degree of the APCN in D_2_O was about the same as that in H_2_O (also about 11). SANS was performed at the Institut Laue-Langevin (ILL)—The European Neutron Source, in Grenoble, France, using the D22 instrument at sample-to-detector distances of 1.4 and 17.6 m, consequently covering a range of *q*-values from 0.0025 to 0.6400 Å^–1^ (DOI: 10.5291/ILL-DATA.EASY-1417). The sample was measured in a 2-mm-thick quartz cuvette. Data reduction was performed using the GRASP software version V.10.21b [45], taking into account the detector effectiveness and transmission of the sample.

## Figures and Tables

**Table 1:** Number-average molar masses, *M*_n_, and molar mass distributions, *Ð*, of the tetraPEG-NH_2_ and tetraPEG-b-OAla-NH_2_ star polymers calculated from their size exclusion chromatograms. Their theoretically estimated molar masses, *MM*_theory_, are also listed in this table.

No.	Sample Name	*MM*_theory_ (g mol^–1^)	Results from SEC	
			*M*_n_ (g mol^–1^)	*Ð*
1	TetraPEG-NH_2_	9996	10,900	1.12
2	TetraPEG-*b*-OAla-NH_2_	11,700	12,700	1.10

## Data Availability

The SANS data can be found at DOI: 10.5291/ILL-DATA.EASY-1417.

## Supplementary Materials

The following supporting information can be downloaded at: https://www.mdpi.com/article/10.3390/gels11050331/s1, Figure S1: ^1^H NMR spectrum of the *N*-carboxy anhydride of *L*-alanine in CDCl_3_. Figure S2. FT-IR spectrum of the *N*-carboxy anhydride of *L*-alanine.
