# Supplementary material for: The First Example of a Model Amphiphilic Polymer Conetwork Containing a Hydrophobic Oligopeptide: The Case of End-Linked Tetra[Poly(ethylene glycol)-b-oligo(L-alanine)]
Source: Gels. 2025 Apr 29;11(5):331. doi: 10.3390/gels11050331 (PMC12111508; doi:10.3390/gels11050331)
Supplement: Supplementary file 1 [file gels-11-00331-s001.zip › gels-3533605-supplementary.pdf]

Supporting Information

The First Example of a *Model* Amphiphilic Polymer Conetwork Containing a Hydrophobic Oligopeptide: The Case of End-linked Tetra[Poly(Ethylene Glycol)-*b*-Oligo(L-Alanine)]

by Demetris E. Apostolides, George Michael, Costas S. Patrickios*, Takamasa Sakai,
Iro Kyroglou, Maria Kasimatis, Hermis Iatrou, Sylvain Prévost and Michael Gradzielski

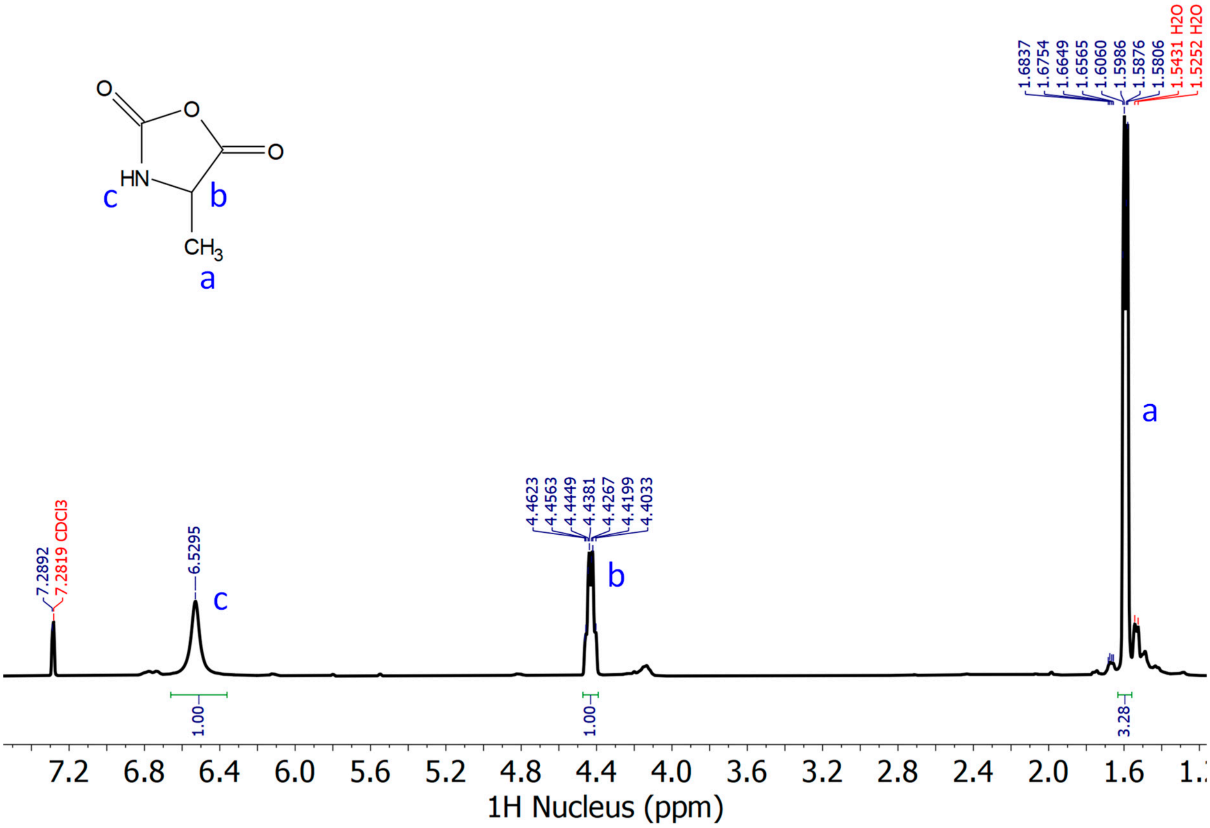

Figure S1. ^{1}H NMR spectrum of the *N*-carboxy anhydride of *L*-alanine in $CDCl_3$.

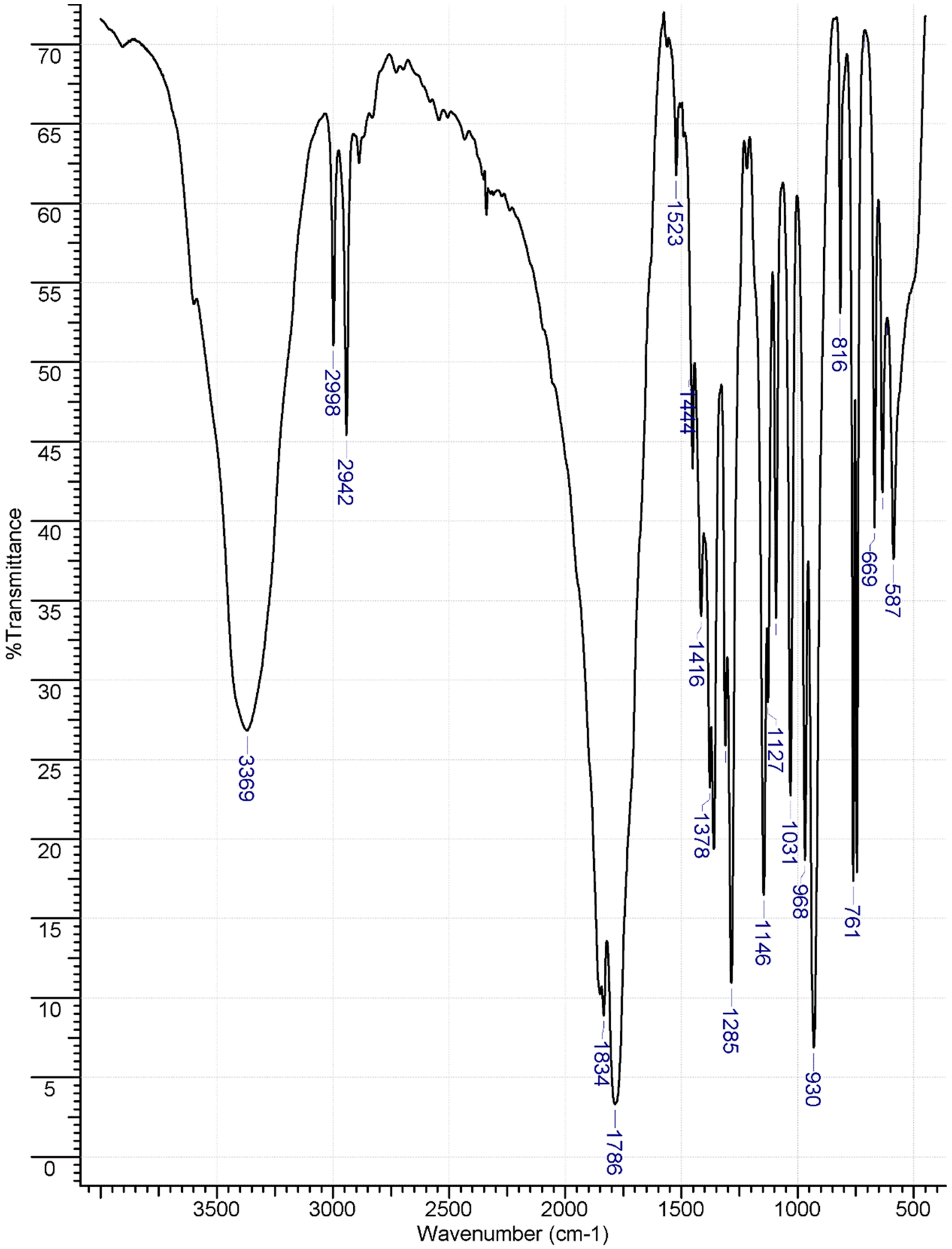

Figure S2. FT-IR spectrum of the *N*-carboxy anhydride of *L*-alanine.
